# Apparent diffusion coefficient as a quantitative biomarker for prostate cancer treatment response on a 1.5 Tesla magnetic resonance-linear accelerator: Impact of image registration and acquisition type

**DOI:** 10.1016/j.phro.2025.100851

**Published:** 2025-10-13

**Authors:** Prashant P. Nair, Joan Chick, Magali Nuixe, Bastien Lecoeur, Yu Xiao, Sian Cooper, Alison C. Tree, Petra J. van Houdt, Uwe Oelfke, Andreas Wetscherek

**Affiliations:** a Joint Department of Physics, The Institute of Cancer Research and The Royal Marsden NHS Foundation Trust, London, United Kingdom; b Department of Computing, Imperial College London, London, United Kingdom; c St John’s College, University of Oxford, Oxford, United Kingdom; d The Royal Marsden NHS Foundation Trust, Sutton, United Kingdom; e Radiotherapy and Imaging Division, The Institute of Cancer Research, London, United Kingdom; f Department of Radiotherapy, the Netherlands Cancer Institute, Amsterdam, Netherlands (the)

**Keywords:** Apparent diffusion coefficient, Repeatability coefficient, Geometric distortion correction, Diffusion weighted echo planar imaging, MR-Linac

## Abstract

**Background and purpose:**

Diffusion-weighted magnetic resonance imaging (DW-MRI) is a quantitative biomarker for cancer detection and treatment monitoring. On magnetic resonance-linear accelerator (MR-Linac) systems, diffusion-weighted echo planar imaging (DW-EPI) suffers from geometric distortion, reducing the repeatability of apparent diffusion coefficient (ADC) measurements. This study evaluated the effect of low-distortion split acquisition of fast spin-echo signal (SPLICE) sequences, and of image registration on the repeatability coefficient (RC) of ADC.

**Materials and methods:**

ADC bias, repeatability, signal-to-noise ratio (SNR) and geometric fidelity were measured in a diffusion phantom using three DW-EPI and two DW-SPLICE protocols. ADC short-term and long-term RCs were measured in healthy volunteers. In patients, the registration of DW-EPI to unweighted images (b0) was tested for its effect on RC in gross tumour volume (GTV) and non-tumour prostate (NT-P), and for its ability to detect significant ADC changes.

**Results:**

Phantom experiments showed strong linear correlation with ground-truth ADC (R^2^ > 0.99). Among EPI protocols, DW-EPI-AP offered the best balance of high SNR and low RC, while Z-direction encoded DW-EPI was the most variable. Both DW-SPLICE variants exhibited reduced distortion compared with EPI but poorer repeatability. In volunteers, long-term RCs (8.0–33.7 %) varied more than short-term RCs (8.9–15.4 %). In patients, registration improved RCs (GTV: 28.0 → 25.1 %; NT-P: 19.6 → 12.6 %) and improved detection of significant ADC change in patients (GTV: 0/6 → 1/6; NT-P: 2/6 → 5/6).

**Conclusion:**

RC and accuracy of DW-EPI agrees with published literature and improves after registration. DW-SPLICE shows lower geometric distortion but would require further optimization and validation to improve repeatability.

## Highlights

• We compared diffusion sequences on a magnetic resonance-linear accelerator (MR-Linac).
• Region of interest size impacts apparent diffusion coefficient (ADC) repeatability.
• Image registration improves ADC repeatability on 1.5 T MR-Linac.
• Registration improves detecting ADC changes during MR-guided radiotherapy.
• Fast spin echo diffusion MRI reduces distortion but requires further optimization.

## Graphical abstract

**Figure:**
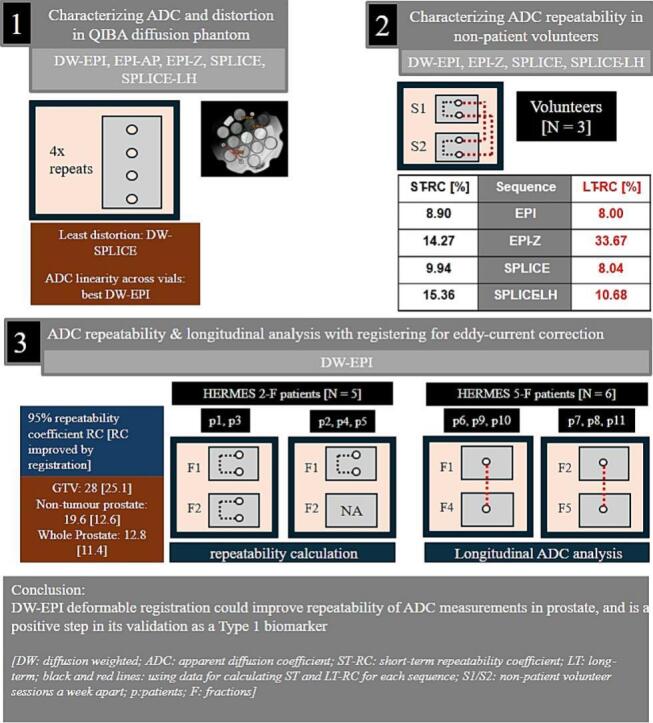

## 1 Introduction

Apparent diffusion coefficient (ADC) maps quantify tissue cellularity in prostate cancer [1,2], distinguishing aggressive and benign tumors [3]. Aggressive tumors, with high cellularity, restrict water movement and lower ADC [[4], [5], [6], [7]]. ADC also serves as a quantitative biomarker for radiotherapy response [[8], [9], [10], [11]], often detecting cellularity changes before lesion size changes [12,13]. Thus, ADC functions both as a disease marker and a longitudinal indicator of treatment response [2].

ADC maps are derived from diffusion weighted (DW) MRI typically using fast echo planar imaging (EPI), to minimize motion effects [14]. DW-MRI however suffers from geometric distortions from off-resonance and eddy currents induced by diffusion-sensitizing gradients [[15], [16], [17]]. At higher b-values, eddy current distortions vary with gradient direction, particularly along phase-encoding, introducing variability in pixel-wise ADC maps and mean values within small ROIs like the gross tumour volume (GTV), especially near boundaries [16].

MR-Linacs integrate functional imaging like DWI with radiotherapy allowing MR-guided adaptive radiotherapy [[18], [19], [20]]. This allows precise tumour localization and daily plan adaptation, improving treatment target coverage and organ-at-risk (OAR) sparing [[21], [22], [23], [24]]. The MR-Linac workflow supports repeated ADC measurements throughout the course of treatment [25]. Clinical use of ADC requires technical validation, including assessment of accuracy and repeatability [26].

The Quantitative Imaging Biomarkers Alliance (QIBA) updated its ADC Profile, defining a >27 % change in mean ADC in prostate lesions as a true biological change with 95 % confidence on 3T scanners [27]. For reliable longitudinal ADC assessment on MR-Linacs, establishing the repeatability coefficient (RC) is essential. A recent study on the 1.5T MR-Linac reported RC of mean ADC in the GTV and other prostate regions of interest [28], while an earlier multiple-fraction prostate study reported an overall increase in ADC across treatment fractions [29].

The first aim of this study was to evaluate ADC bias and repeatability between low-distortion DW-sequences against a baseline DW-EPI sequence in a diffusion phantom and healthy volunteers. The second aim was to evaluate the impact of registering DWIs to the b0 image on ADC repeatability of pixel-wise fit ADC and present longitudinal data relative to the calculated ADC repeatability coefficient.

## 2 Materials and methods

### 2.1 Imaging protocols

DWI data were acquired on a Unity 1.5T MR-Linac (Elekta AB, Stockholm, Sweden). The baseline protocol (DW-EPI) used b-values of 0, 30, 150, and 500 s/mm^2^, based on the consensus protocol [30]. Additional protocols acquired in phantoms and non-patient volunteers were:

• DW-SPLICE [31,32], SPLICE is a single-shot diffusion-sensitized TSE sequence using split-echoes for improved SNR. In addition to conventional linear ordering, we tested a variant with centre-out ordering of phase-encoding steps (DW-SPLICE-LH).
• DW-EPI-Z uses diffusion encoding along the scanner’s Z-axis to reduce misregistration [30] by avoiding direction-dependent distortions and X/Y gradient-induced eddy currents.
• Another DW-EPI protocol with phase encoding in the anterior-posterior direction [DW-EPI-AP] was performed only in the phantom.

3D T2-weighted images (T2W) were acquired as distortion reference in the phantom, and for contour-delineation in patients. See Table S1 for details.

### 2.2 Phantom validation

A diffusion phantom (Qalibre MD Inc., Boulder, CO, USA) [33] was scanned axially and prepared as described in other works [25,27,[34], [35], [36]]. The phantom was kept approximately at 0° C using an ice-water bath [37].

Analysis was performed using qCal-MR® (Caliber MRI, Boulder, CO, USA), an automated quantitative MRI QC software (details in Supplementary). Signal-to-noise ratio (SNR) and Short-term repeatability (ST-RC)were averaged across the original experiment and follow-up performed after a year (Repeat 1 and Repeat 2) for each protocol. Individual session values are detailed in the Supplementary Material. ADC bias and linearity were assessed by comparing measured ADC values to ground truth values from the phantom manual using linear regression using MATLAB (R2023a, The MathWorks, Natick, MA, USA). Bias was considered evident if the 95 % CI of the slope did not include 1 or the intercept did not include 0. Geometric distortion was assessed on b-value 500 s/mm^2^ images by manually measuring distances between phantom markers (Fig. 2). Measurements were repeated five times on the central slice to address challenges in locating the marker centre, particularly in DW-EPI sequences. Distances were compared against T2W images using a paired *t*-test. A p-value below 0.05 was considered evidence for statistical significance.

**Fig. 1:**
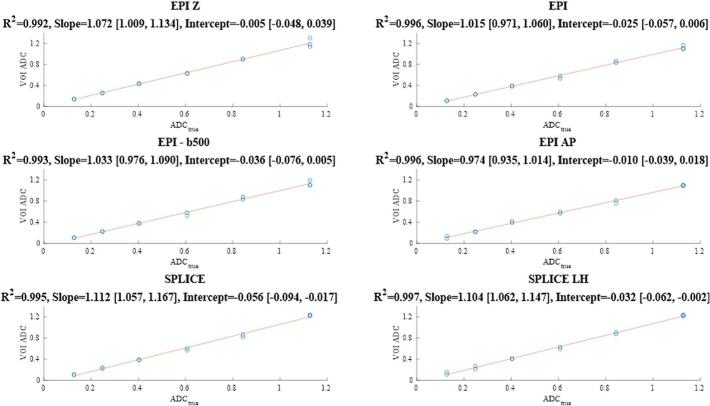
Linear fits of measured versus reference ADC values for each sequence are shown in 10^−3^ mm^2^/s, with R^2^, slope (proportional bias; ideal = 1), and intercept (additive bias; ideal = 0) reported alongside ±95 % CIs. All sequences exhibit strong linearity (R^2^). DW-EPI and DW-EPI-AP show no detectable bias. DW-EPI-Z demonstrates proportional but not additive bias. DW-SPLICE performs the worst, exhibiting both additive and proportional biases. EPI-b500 refers to ADC calculated the b-values of 0 and 500 s/mm^2^, unlike the others where ADC was derived using all the b-values.

**Fig. 2:**
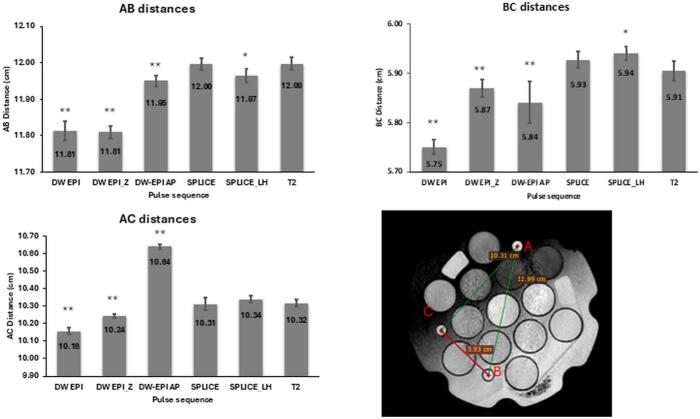
Distances between markers in the phantom measured on various DW images and 3D T2W images, which served as distortion-free reference. All DW-EPI sequences caused significant distortion compared to the reference for all the marker distances, DW-SPLICE-LH showed reduced distortion, while DW-SPLICE showed no significant distortion compared to the T2. Values are represented as mean ± SD; * indicates p < 0.05; ** indicates p < 0.01.

### 2.3 Volunteer validation

We scanned prostate of three male healthy volunteers (ages 29, 35 and 43), who consented to participation in the PRIMER trial (REC 17/LO/0907). Each volunteer was scanned twice to assess ADC repeatability. Long-term (LT) ADC repeatability was assessed between two sessions acquired one week apart (A, B) and short-term (ST) ADC repeatability was tested in the same session without repositioning. ST-RC was assessed using scan pairs acquired within the same session: (A.1, A.2) for Session A and (B.1, B.2) for Session B. Long-term repeatability (LT-RC) was evaluated using pairs acquired across sessions, one week apart: (A.1, B.1) and (A.2, B.2). Each repeatability metric was calculated using six such scan pairs. To assess statistical power of the comparisons with six scan-pairs, a Monte-Carlo-based power analysis was conducted.

ADC bias for the experimental protocols and DW-EPI is reported as the average ADC and the delta ADC between each pair of ST and LT repeats using Bland-Altman plots. Paired t-tests were used to assess differences in ADC values measured with each sequence. A p-value below 0.05 was interpreted as evidence of statistical significance.

### 2.4 Patient studies

This retrospective study included data from eleven prostate cancer patients treated on our MR-Linac, who consented to the HERMES clinical trial (REC 20/LO/1162) which randomised between two-fraction (2#) or five-fraction (5#) treatments [38]. The median[min–max] age of the patients was 72 (53–77) years. All patients received neoadjuvant and concurrent androgen deprivation hormone therapy (ADT). All patients were positioned head-first supine using the 8-channel posterior/anterior coil of the MR-Linac.

Seven treatment fractions (where raw data was available) from five 2# patients [P1–P5] were analysed to calculate RC. Patients were instructed to empty their bladders and were repositioned between the test and retest scans in the same fraction. Structural images, used for contouring GTV and OAR, were reacquired after repositioning.

Two datasets each from six 5# patients [P6–P11], acquired one week apart, were analysed to detect true treatment responses based on RC established from the 2# patients.

#### 2.4.1 Image registration

DW-images corresponding to individual diffusion weightings and directions were reconstructed in the Philips pulse programming environment (Paradise R5.7.1, Philips Medical Systems, Best, The Netherlands). To mitigate eddy current-related geometric distortions, we used multi-resolution B-spline registration [39], performed using a custom Python package built on the ITK/Elastix framework. The DW-images from each orthogonal DW direction were registered to the corresponding b0 image. Details are provided in Supplementary Material. We evaluated the impact of registering differently distorted DW-direction images onto the b0, on ADC bias and repeatability.

#### 2.4.2 Image processing

DICOM images were exported for both registered and non-registered workflows and ADCs were calculated using all b-values in MATLAB. A body mask [40] was applied to the DW-images and a linear model was fitted to the logarithm of the signal intensities:

$$\ln\left(S\left(b\right)\right)=\ln\left(S(b0)\right)-b\times ADC$$

For each patient, GTV and whole prostate contours were propagated onto test and retest ADC maps from the corresponding structural images on Ray Station (2024A, RaySearch Laboratories AB, Stockholm, Sweden). A non-tumour prostate (NT-P) ROI was drawn later, adjacent and size-matched to the GTV to avoid confounding effects, as prior work [29] reported a steep increase in RC for volumes below 2 cm^3^. ADCs were computed over the full contour mask, including contributions from both fully enclosed and partial (boundary) pixels.

#### 2.4.3 Statistical analysis

Scatter plots were used to visualize ADC values for non-registered and registered images of the GTV (1.08 ± 0.74 cm^3^) and NT-P (0.84 ± 0.45 cm^3^) for the 2# data. Paired t-tests were used to compare registered and non-registered ADCs for each structure and to compare ADC values of the GTV with NT-P and whole-prostate (WP). To assess statistical power of the comparisons with seven scan-pairs, a Monte-Carlo-based power analysis was conducted.

ADC RCs were calculated as described by QIBA [41] on 2# data for GTV, NT-P and WP using the formula.

$$\mathrm{RC}=1.96\times \sqrt{2}\times within-subject\;standard\;deviation.$$

Longitudinal ADC analysis was performed on 5# ADC data for both registered and non-registered images on GTV (0.41 ± 0.34 cm^3^) and NT-P (0.28 ± 0.19 cm^3^). 2# RCs were used as 95 % confidence intervals to differentiate true physiological change from measurement error in the 5# cohort.

## 3 Results

### 3.1 Phantom validation

The temperatures of the ice-water bath before and after the experiments were 0.01 and 0.04 °C, respectively.

All sequences demonstrated high R^2^ values (0.992–0.997), indicating strong linear correlation between measured ADC and ground truth. DW-EPI-Z had evident proportional bias but no additive bias. DW-EPI and DW-EPI-AP had neither additive nor proportional biases. Averaging across sessions, EPI-AP showed the highest SNR (105) with low ST-RC (12 × 10^−6^ mm^2^/s), remaining within the 15 × 10^−6^ mm^2^/s tolerance. SPLICE-LH demonstrated the highest SNR among the SPLICE variants (51) but repeatability was poor (ST-RC = 42). Among the EPI protocols, EPI-Z achieved lower SNR (56) with the highest variability (ST-RC = 25), while standard EPI yielded intermediate SNR (68) with comparable repeatability (ST-RC = 19). SPLICE showed similar SNR to SPLICE-LH (51) but the poorest repeatability overall (ST-RC = 50) compared to SPLICE-LH’s 42. (See Supplementary A2.1 and Table S3 for further details on long-term repeatability).

The DW-EPI sequences showed evidence of differences in distances between markers A, B, and C compared to the T2W image (p < 0.01). DW-SPLICE-LH exhibited reduced distortion, with evidence of differences in distances AB and BC (p < 0.05), but not for distance AC. For DW-SPLICE, there was no evidence of distortion compared to the T2W image (Fig. 2).

### 3.2 Volunteer validation

ST‑RC and LT‑RC for DW‑EPI were 112 (8.9 %) and 97 (8.0 %) × 10^−6^mm^2^/s, respectively. Compared with DW‑EPI, ST‑RCs were higher for DW‑SPLICE (137, 9.9 %), DW‑SPLICE‑LH (216, 15.4 %), and DW‑EPI‑Z (255, 14.3 %), while LT‑RCs increased for DW‑SPLICE (109, 8.0 %), DW‑SPLICE‑LH (150, 10.7 %), and DW‑EPI‑Z (601, 33.7 %). Statistical power analyses confirmed adequate power (>0.8) for ST‑RC comparisons across sequences, whereas LT‑RC comparisons had moderate power for DW‑SPLICE (0.72) but were sufficiently powered for the other sequences (Tables S7–S8).

**Fig. 3:**
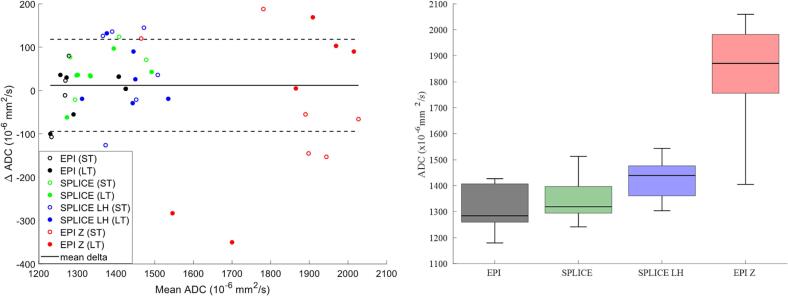
Delta ADC measured in the prostate of three healthy volunteers for different DW sequences (colour-coded). Hollow markers denote short-term repeatability (within the same session). Filled markers denote long-term repeatability between sessions A and B (one week apart) across DW sequences for the three volunteers. Average delta ADC and ±1 SD are shown. The bar graphs show ADC values across sequences. The EPI-Z sequence exhibits significantly higher ADCs compared to all other sequences. DW-SPLICE-LH shows significantly higher ADCs than DW-EPI and DW-SPLICE, between which no significant difference is observed.

DW-EPI-Z had a significant positive ADC bias compared to other sequences (mean: 1833 × 10^−6^ mm^2^/s, p < 0.01). ADC values from DW-SPLICE-LH (1426 × 10^−6^ mm^2^/s) were significantly higher than those from DW-SPLICE (1348 × 10^−6^ mm, p < 0.01). No significant difference was observed between DW-EPI (1313 × 10^−6^ mm^2^/s) and DW-SPLICE (Fig. 3).

### 3.3 Patient studies

**Fig. 4:**
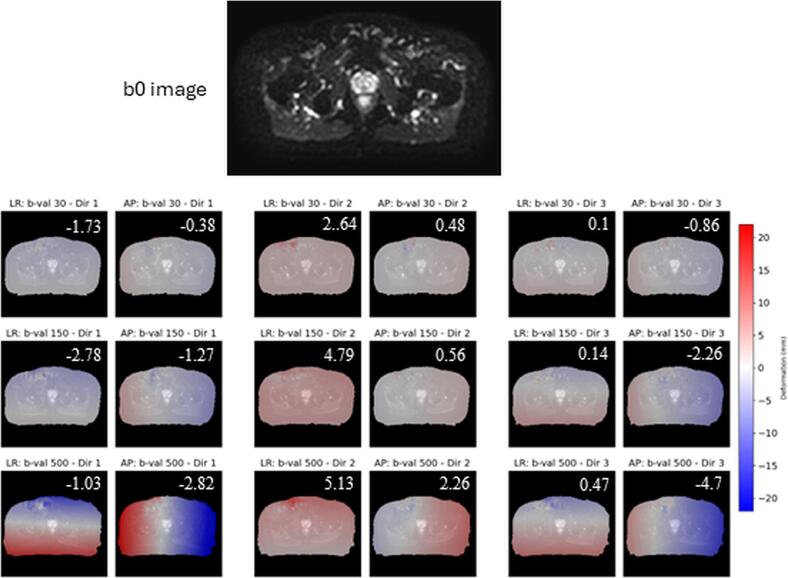
Registration results for a slice containing the GTV anatomy from patient P11. The non-deformed b0 image (fixed) is shown along with deformation fields in the left–right (LR) and anterior–posterior (AP) directions for each b-value and diffusion encoding direction, overlaid on the corresponding diffusion image (moving images). Numbers indicate the average deformation (mm) within the prostate contour. For each diffusion-weighting direction, the scanner’s three physical axes (X, Y, Z) contribute: Dir 1 = [−0.67, 0.33, −0.67], Dir 2 = [0.33, −0.67, −0.67] and Dir 3 = [−0.67, −0.67, 0.33]. Globally, deformation is higher for higher b-values. Deformations for diffusion direction 2 follows an opposite pattern to that of the deformations in diffusion encoding 1 and 3.

Compared to the b0 image, deformations increased globally with higher b-values and varied by direction; WP contour displacements reached up to 5  mm (Fig. 4).

**Fig. 5:**
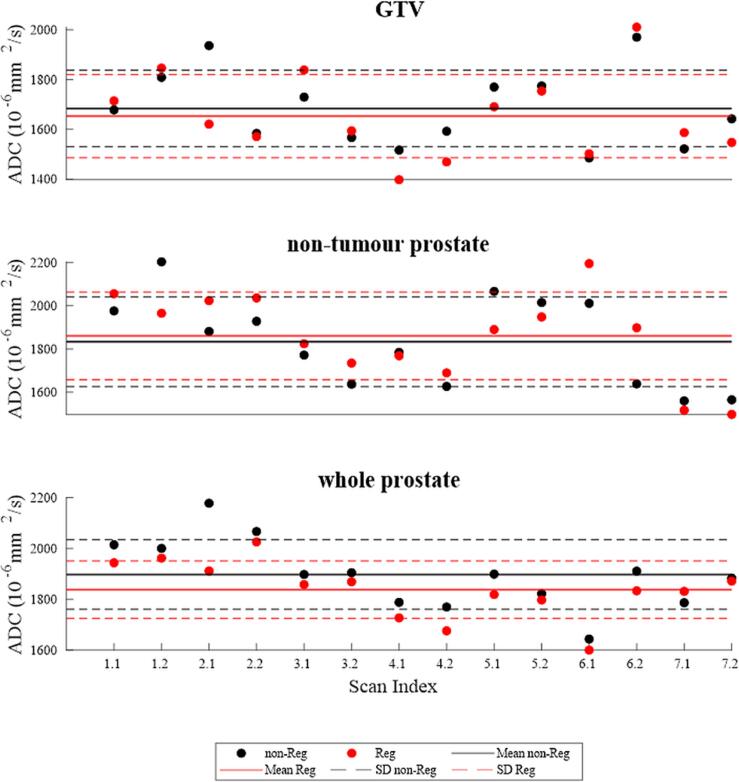
Mean ADC for the GTV, non-tumour prostate (NT-P) and whole prostate (WP) in the 14 scans from the 7 fractions used to calculate the RC. Registration reduced the ADC in the whole prostate. The ADC in the GTV is significantly lower than for NT-P for both the registered (Reg) and the non-registered (non-Reg) workflows.

The mean (±SD) time between the repeats in 2# scans was 89.8 ± 8.2 min. Mean ADC values averaged across scans for GTV, and WP were 1684, 1833 and 1897 × 10^−6^ mm^2^/s. While registration did not significantly change the mean ADCs in the GTV (1653 × 10^−6^ mm^2^/s, p = 0.3) and NT-P (1860 × 10^−6^ mm^2^/s, p = 0.47), it reduced the mean ADC in WP (1837 × 10^−6^ mm^2^/s, p < 0.01). Mean ADC in the GTV was significantly lower than in NT-P for both the non-registered (p = 0.03) and registered (p = 0.005) workflows (Fig. 5).

RC calculation: Absolute RCs in 10^−6^ mm^2^/s (relative RC) were 483 (28.0 %), 362 (19.6 %) and 234 (12.8 %) for GTV, NT-P and WP, respectively. Registration improved these values to 438 (25.1 %), 251 (12.6 %), and 200 (11.4 %). Statistical power analyses confirmed that the improvement was significant in NT-P (power = 0.94) and moderate power for GTV (0.71) and WP (0.77). (Table S9).

**Fig. 6:**
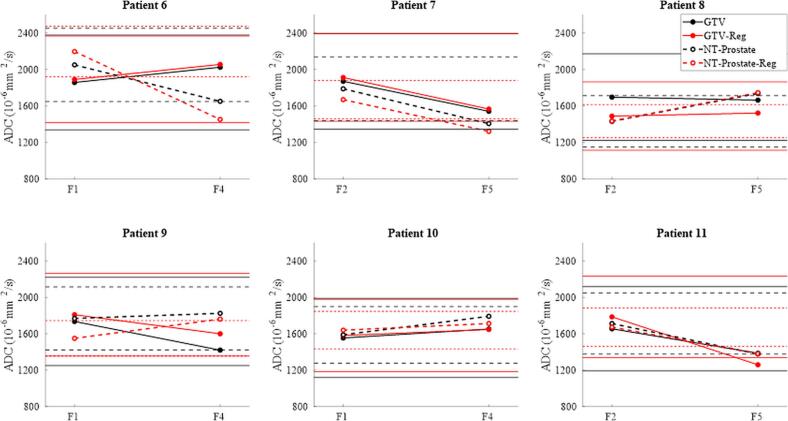
ADC measurements for six patients in the GTV and non-tumour prostate (NT-P) from the five-fraction cohort, comparing non-registered (black) and registered (red) data. Measurements were taken at two different fractions (F), roughly one week apart. Dashed lines and hollow circles represent NT-P while bold lines and filled circles represent GTV. Horizontal lines represent 95% confidence limits for detecting a true change. (For interpretation of the references to colour in this figure legend, the reader is referred to the web version of this article.)

Longitudinal ADC analysis: No significant ADC change (>95 % confidence) was detected in the GTV for patients 6–10, regardless of whether registration was used. In patient 11, a significant change was observed only when images were registered. For NT-P, a significant ADC change was observed in patients 7 and 8, while the registered workflow detected significant change in all patients except patient 10 (Fig. 6).

## 4 Discussion

We evaluated ADC repeatability and bias on a 1.5T MR-Linac using NIST/QIBA phantom and healthy volunteer data, comparing low-distortion DW-SPLICE with DW-EPI. Although DW-SPLICE improved geometric fidelity in the phantom, it did not enhance repeatability in volunteers. Distortion correction by registering diffusion images to the b0 prior to trace-weighted image calculation, which improved RC and may increase confidence in attributing ADC changes to physiological effects.

In the phantom, DW-EPI-AP, DW-SPLICE, DW-SPLICE-LH and DW-EPI-Z were compared to the baseline DWI-EPI. The ADC values from all sequences had high linearity across the range of values in the phantom’s vials, indicated by high R^2^ values from the linear regression analysis between ground truth and measured ADC values.

The EPI-based methods (DW-EPI, DW-EPI-b500, and DW-EPI-AP) gave the most accurate measurements, without evident proportional and additive biases (Fig. 1). Averaged across the original and follow-up 2 sessions, DW-EPI-AP provided the highest SNR with consistently low RC, while DW-EPI-Z achieved moderate SNR with the highest variability, and standard DW-EPI yielded intermediate SNR with comparable repeatability. Among the SPLICE-based methods, DW-SPLICE and DW-SPLICE-LH achieved SNR values similar to each other (∼51), but both exhibited substantially poorer repeatability than all EPI-based protocols.

Further, repeated phantom experiments overall revealed marked variability between follow-up sessions (after ∼1 year). While EPI RC improved in the first follow-up, this effect was not reproduced consistently in later sessions after a month, highlighting instability in phantom-based SNR and RC measurements over time (Supplementary Section A-2).

From the three volunteer dataset, we calculated short-term RC to assess machine-related variability under controlled conditions, and long-term RC from repeated scans one week apart. Long-term RC is clinically more relevant, capturing repositioning and physiological changes, consistent with prior clinical repeatability study designs [42].

Long-term repeatability was comparable to short-term repeatability, except for DW-EPI-Z. Physiological variations over one week remained within measurement variability for DW-EPI and DW-SPLICE, although the limited sample size (six pairs) is well below the 35 required for a formal claim [27] − our goal was to compare RCs across sequences under controlled conditions (same subjects) using the RC as a metric. The DW-EPI baseline sequence on our 1.5T MR-Linac showed short-term and long-term repeatability of 8.9 % and 8.0 %, respectively, compared to 10 % repeatability and 19 % reproducibility previously reported [28]. The differences in the latter may thus be reflecting treatment effects; however, further validation in a larger cohort is required. Our data suggest DW-EPI is more repeatable than SPLICE-LH and EPI-Z, and that SPLICE shows no repeatability advantage over EPI [Tables S6 and S7].

DW-SPLICE did not outperform baseline DW-EPI in RC, suggesting geometric fidelity alone may not ensure improved in-vivo repeatability. An optimised 7-minute DW-SPLICE improved RC [43], but shorter scans suit RT workflows. Despite low distortion aiding delineation [44], lesions were often not visible on DWI, requiring T2-based contours. DW-SPLICE may benefit from tissue-specific SNR optimisation [45].

For the patient studies, DW‑EPI consensus protocol, (section 2.1) was selected due to superior ADC repeatability in volunteers. To address distortion‑induced misregistration between diffusion‑weighted and b0 images, a deformable registration step was applied, which did not significantly alter mean ADC in GTV or NT-P, consistent with prior findings showing minimal ADC impact but potential delineation benefits [46]. In two-fraction patients, GTV ADC values were significantly lower than NT-P, independent of registration and remained valid despite neoadjuvant and concurrent ADT. These findings are consistent with a previous MR-Linac study showing lower tumour ADC compared to non-tumour prostate tissue [29]. Together, these results support the role of ADC as a Type 0 quantitative imaging biomarker [47] on the MR-Linac.

The split gradient coil design of the Unity MR-Linac makes eddy-current correction important for diffusion-weighted MRI [30]. We corrected distortions by registering DWIs to b0, though readout distortions remained uncorrected without reverse-phase b0 [46]. Deformable registration reduced intra-subject variability and improved ADC repeatability in NT-P (RC: 19.6 % to 12.6 %) but less marked in GTV (28 % to 25.1 %).This likely reflects the ADC measurement sensitivity in small structures, where many boundary pixels neighbour different tissues, making measurements more prone to deformation effects.

Longitudinal ADC changes may reflect true physiological changes if exceeding ±95 % RC [48], derived from 2# repeat scans within one hour. Despite expected baseline comparability [38], smaller GTV and NT-P volumes in the 5# arm may reduce ADC reliability. Fig. 6 shows higher change-detection confidence in NT-P, further improved by registration, with marginal effect in GTV. However, we did not observe the previously reported trend of increasing ADC over time [29]. A limitation is having only two time points per patient in a hypo-fractionated setting with concurrent ADT. Nonetheless, our aim was to present longitudinal data relative to the calculated RC and to establish a framework for identifying physiologically meaningful ADC change, supporting progress towards validation of ADC as a Type 1 quantitative biomarker [47].

A limitation of ADC calculation in very small GTVs and NT-Ps is the high proportion of boundary pixels, which may introduce variability in mean ADC [27]. This effect appears more pronounced on the MR-Linac, where prostate deformation reached two to three pixels, (Fig. 4), with greater impact on NT-P than GTV. While increased sample size may stabilise these findings, RC estimates remain sensitive to limited data [27]. Our aim here was to highlight the potential impact of the ROI-size/shape and varying with b-value and DW- direction on ADC calculation. For patient P11 the deformations (Fig. 4) significantly impacted GTV delta ADC across fractions (Fig. 6).

Another limitation is that RC test–retest scans calculation were acquired during the same fraction, one prior to and one after radiation, potentially introducing treatment-related confounders. Ideally, both should precede treatment [49]. For multi-institute processing, storing individual DW images before averaging is also recommended.

To summarize, we assessed the repeatability of the ADC and geometric distortion using the NIST/QIBA Diffusion Phantom and in healthy volunteers for different DWI protocols. The DW-SPLICE and DW-SPLICE-LH images exhibited minimal distortion in the phantom, but not in healthy volunteer experiments. Our results from patient volunteers scanned with DW-EPI suggest that use of deformable registration to mitigate eddy-current-related distortions could improve repeatability of ADC measurements within the prostate, which is a positive step in its evaluation as a Type 1 biomarker. Larger patient cohorts and multi-centre data would be required to confirm these findings.

## Declaration of Generative AI and AI-assisted technologies in the writing process

During the preparation of this work the authors used chatGPT in order to reduce word count. After using this tool, the authors reviewed and edited the content as needed and take full responsibility for the content of the publication.

## CRediT authorship contribution statement

**Prashant P. Nair:** Conceptualization, Formal analysis, Methods, Visualization, Original Draft, Writing - review and editing. **Joan Chick:** Formal analysis, Visualization, Writing - review and editing. **Magali Nuixe:** Visualization, Writing - review and editing. **Bastien Lecoeur:** Methods, Writing - review and editing. **Yu Xiao:** Methods, Writing - review and editing. **Sian Cooper:** Formal Analysis, Writing - review and editing. **Alison C. Tree:** Resources, Writing - review and editing. **Petra J. van Houdt:** Methods, Writing - review and editing. **Uwe Oelfke:** Resources, Writing - review and editing. **Andreas Wetscherek:** Conceptualization, Formal Analysis, Resources, Methods, Supervision, Visualization, Writing - review and editing.

## Declaration of competing interest

The authors declare the following financial interests/personal relationships which may be considered as potential competing interests: PPN, JC, MN, BL, SC, ACT, PJvH, UO and AW declare the following conflict: The Institute of Cancer Research (ICR), the Royal Marsden Hospital (RMH) and the Netherlands Cancer Institute (NKI) are members of the MR-Linac Consortium with industrial partners Elekta and Philips. ICR and RMH receive research support from Elekta and Philips. ACT receives research funding from Elekta, Varian and Accuray, honoraria/travel assistance from Elekta, Accuray, Bayer and Janssen. ACT is chair of the MR linac consortium steering committee.
