# Supplementary material for: Apparent diffusion coefficient as a quantitative biomarker for prostate cancer treatment response on a 1.5 Tesla magnetic resonance-linear accelerator: Impact of image registration and acquisition type
Source: Phys Imaging Radiat Oncol. 2025 Oct 13;36:100851. doi: 10.1016/j.phro.2025.100851 (PMC12554063; doi:10.1016/j.phro.2025.100851)
Supplement: Supplementary Data 1 [file mmc1.docx]

# Supplementary A: Phantom Studies

## A-1: Sequence parameters

The DW-EPI pulse sequence is based on the HERMES protocol used to scan the volunteers and the patients. The other DW-EPI protocols are modified from this.

1. DW-EPI-AP: the phase encoding is altered to the anterior-posterior direction.
2. DW-EPI-Z: diffusion is only encoded in the Z direction.

Table S1:Sequence parameters

| Sequence | DW-EPI [baseline] | DW-EPI -Z | SPLICE | SPLICE - LH |
| --- | --- | --- | --- | --- |
| b-values (averages) (s/mm^2^) | 0 (6), 30 (6), 150 (6), 500 (14) | 0 (6), 30 (6), 150 (6), 500 (14) | 0 (2), 150 (4), 500 (12) | 0 (2), 150 (4), 500 (12) |
| TR (ms) | 2960 | 3079 | 3000 | 3000 |
| TE (ms) | 82 | 88 | 91 | 73 |
| Fat suppression | SPAIR | SPAIR | SPAIR | SPAIR |
| Flip angles (°) | 90 | 90 | 90 | 90 |
| TSE / EPI factor | 47 | 47 | 32 | 32 |
| SENSE | 2.3 | 2.3 | 2 | 2 |
| PE direction | Right-Left | Right-Left | Right-Left | Right-Left |
| Half scan factor | no | no | 0.6 | 0.6 |
| FOV (mm) | 430, 430 | 430, 430 | 430, 430 | 430, 430 |
| Matrix | 108, 108 | 108, 108 | 108, 106 | 108, 106 |
| Pixel size (mm) | 3.98, 3.98 | 3.98, 3.98 | 3.98, 4.06 | 3.98, 4.06 |
| Recon pixel size (mm) | 1.92 | 1.92 | 1.92 | 1.92 |
| Slice thickness (mm) | 4 | 4 | 4 | 4 |
| Nr. slices | 15 | 15 | 15 | 15 |
| TSE Echo spacing (ms) | NA | NA | 3.9 | 4.9 |
| Bandwidth (Hz) | 32.9 | 32.9 | 1447 | 870.2 |
| Acquisition time (min: seconds) | 4:12 | 1:42 | 5:00 | 5:00 |

## A-2: QCAL Analysis

## A-2.1: Generation of Data Structures and Protocol compliance with QIBA phantom profile

As per QibaPhan SW release 1.0x64 guidelines, the following workflow was used to ensure consistent and repeatable ADC quantification across all DW protocols:

1. Data Acquisition

Each DW protocol was acquired in four passes on the QIBA phantom. DICOM images were exported into a “DICOM” folder.

1. Software Setup

QibaPhan software was installed, and the first module (qiba_mbuild.exe) was initiated via command line. The software automatically sorted images by series and protocol, generating image_order.mat files and organizing data into protocol-specific folders. The software displayed a scan catalogue and created labelled DWI structures (“Passes” and “Sets”), saved in a timestamped *DataStructsBin folder.

1. Protocol Compliance Check

A master protocol file was used to check compliance with QIBA acquisition standards. Deviations were summarized (Table S2) but not adjusted, as our aim was to benchmark clinically optimized protocols on the MR-Linac rather than to conform to the QIBA profile.

1. ADC and SNR Map Generation

ADC maps and temporal SNR maps were automatically generated and stored in the *DataStructsBin folder. For each ROI, SNR was computed by dividing the mean signal of the averaged b0 image (across repeats) by the mean signal of the noise image (standard deviation across repeats).

1. ROI Definition and Verification

ROI generation was semi-automated. Circular ROIs (10 mm diameter) were manually centred on each vial using the first pass ADC image for three centre slices, then propagated across all passes.

1. ROI statistics

ROI statistics (mean, median, SD) and multi-pass SNR values were computed and saved as binary files, CSV tables, and PDF montages in the ResultsQCstats folder.

Table S2: Protocol non-conformance to QIBA profile for best ADC performance

| Parameter | QIBA Profile | DW-EPI [baseline] | DW-EPI Z | DW-SPLICE | DW-SPLICE LH |
| --- | --- | --- | --- | --- | --- |
| TR (ms) | 7000 - 10000 | 2960 | 3080 | 3000 | 3000 |
| Pixel bandwidth (Hz) | 1000 - 2500 | 2540 | 2540 | 1447- conforming | 870 |
| Matrix | 128 - 160 | 108 | 108 | 108 | 108 |
| FOV (mm) | 210 – 230 | 430 | 430 | 430 | 430 |
| b-values (s/mm^2^) | [0 500 1000 1500 2000] | [0 30 150 500] | [0 30 150 500] | [0 150 500] | [0 150 500] |
| SENSE factor | 1.9 – 2.1 | 2.3 | 2.3 | 2 - conforming | 2 - conforming |

Table S3: Metrics for the central vial:- SNR (b0), ADC bias (absolute and %) and ADC RC (absolute and %).

| **Phantom study** | **Protocols** | **SNR (QCal)** | **SNR efficiency** | **ADC Bias**  **(* 10-6 mm2/s)** | **ST-RC (* 10-6 mm2/s) [calculated 4-pass]: Threshold = 15.0** | **LT-RC (* 10-6 mm2/s) [calculated from pass 1s]** |
| --- | --- | --- | --- | --- | --- | --- |
| Original Experiment | EPI | 35 | 8.31 | -31.7 (-2.8%) | 33 | - |
|  | EPI AP | 38 | 9.02 | -48.0 (-4.2%) | 22 | - |
|  | EPI Z | 40 | 9.31 | 13.5 (1.2%) | 36 | - |
|  | SPLICE | 56 | 22.86 | 88.3 (7.8%) | 44 | - |
|  | SPLICE LH | 77 | 31.44 | 85.1 (7.5%) | 8 | - |
| Follow-up Repeat1*,+ | EPI | 200 | 47.46 | -55.6(-4.9%) | 0.4 | - |
|  | EPI AP | 113 | 26.81 | -48.2(-4.2%) | 4.5 | - |
|  | EPI Z | 137 | 31.87 | -47.5(-4.2%) | 12.0 | - |
| Follow-up Repeat2*,+ | EPI | 105 | 24.92 | -53.4(-4.7%) | 8.1 | 3.6 |
|  | EPI AP | 107 | 25.39 | -35.5(-3.1%) | 7.2 | 30.99 |
|  | EPI Z | 125 | 29.08 | -43.0(-3.8%) | 5.5 | 19.44 |
| Follow-up 2  Repeat1 | EPI | 67 | 15.90 | -62.7(-5.6%) | 12.67 | - |
|  | EPI AP | 99 | 23.49 | -42.8(-3.8%) | 3.71 | - |
|  | EPI Z | 70 | 16.29 | -30.8(-2.7%) | 21.86 | - |
|  | SPLICE | 44 | 17.96 | 68.1(6.0%) | 51.24 | - |
|  | SPLICE LH | 36 | 14.70 | 16.5(1.5%) | 68.58 | - |
| Follow-up 2  Repeat 2 | EPI | 101 | 23.97 | -31.5(-2.8%) | 10.07 | 22.66 |
|  | EPI AP | 177 | 42.00 | -44.47(-3.9%) | 8.81 | 3.26 |
|  | EPI Z | 59 | 13.73 | -18.5(-1.6%) | 18.47 | 13.13 |
|  | SPLICE | 54 | 22.05 | 21.1(1.9%) | 54.02 | 89.15 |
|  | SPLICE LH | 39 | 15.92 | 61.4(5.4%) | 50.04 | 103.34 |
| Averaged measures across original and Follow-up 2 | EPI | 67.67 | 16.06 | -41.9(-3.72%) | 18.58 | - |
|  | EPI AP | 104.67 | 24.84 | -45.0(-4.0%) | 11.51 | - |
|  | EPI Z | 56.33 | 13.11 | -11.93(-1.1%) | 25.44 | - |
|  | SPLICE | 51.33 | 20.96 | 59.17(5.25%) | 49.75 | - |
|  | SPLICE LH | 50.67 | 20.68 | 54.33(4.82%) | 42.21 | - |

1. Original experiment: Phantom scans across all sequences showed that EPI and its variants exceeded the repeatability coefficient (RC) threshold of 15 × 10⁻⁶ mm²/s, while SPLICE sequence had higher SNR but also larger bias and RC values.
2. Follow-up 1 (after ~1 year): Repeated phantom scans of EPI variants showed markedly higher SNR values compared to baseline and, importantly, all EPI sequences fell within the 15 × 10⁻⁶ mm²/s RC threshold. SPLICE was not repeated due to licensing restrictions.
3. Follow-up 2 (after ~1 month): Both EPI and SPLICE sequences were repeated, as we obtained the licences from the vendor in this time. SNR values varied substantially across repeats, with SPLICE showing markedly lower SNR and poorer RC behaviour.
4. Intra-session repeatability (Follow-up 2, two repeats): Notable variability was observed between repeat 1 and repeat 2 scans, particularly for SNR and RC values across both EPI and SPLICE. We believe that consistency in phantom positioning, alignment with the isocentre and coil elements perhaps might improve reproducibility.
5. SNR efficiency is computed as follows

$$\eta=SNRmeas/\surd(nAvgs\left( b0 \right)*time\left( single b0 \right))$$

Where time of single b0 is the TR (ignoring the hardware overheads)

1. Overall observations: While EPI sequences appeared to improve in repeatability between baseline and follow-up 1, this improvement was not consistent across subsequent repeat experiments. Importantly, SPLICE demonstrated lower SNR than EPI, and less stable repeatability behaviour.

Further, in the original experiment, all sequences demonstrated high R^2^ values (0.992-0.997), indicating strong linear correlation between measured ADC and ground truth. DW-EPI-Z had evident proportional bias but no additive bias. DW-EPI and DW-EPI-AP had neither additive nor proportional biases.

As shown in Fig. S1, during follow-up 2, all the sequences demonstrated R^2^ values (0.97 – 0.994), indicating marginally lower linear correlation compared to the original session. None of the sequences had a proportional bias. However, both EPI-AP and EPI Z had evident additive bias.


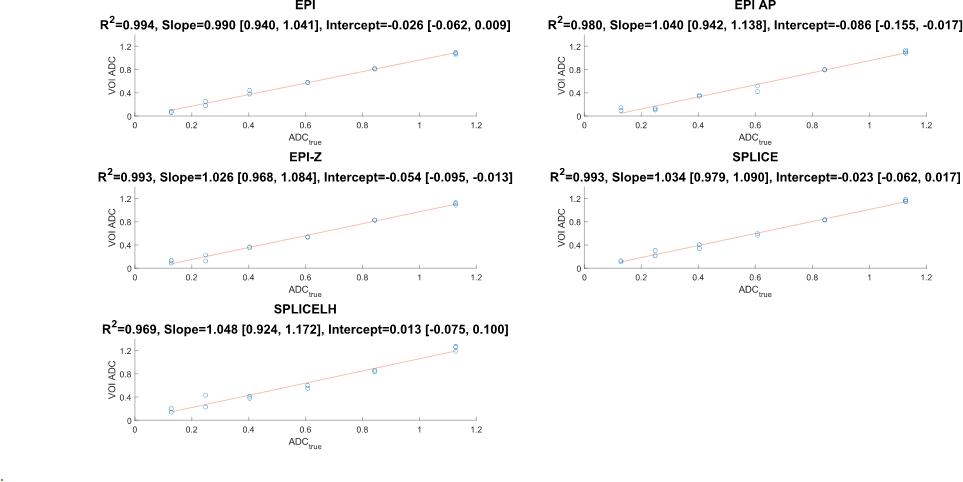


Figure S1: Linear fits of measured versus reference ADC values for each sequence are shown in 10^-3^mm^2^/s, with R2, slope (proportional bias; ideal = 1), and intercept (additive bias; ideal = 0) reported alongside ±95% CIs.

# Supplementary-B: Image Registration

To correct distortions caused by eddy currents in diffusion-weighted (DW) imaging, we used a slice-by-slice registration approach that aligns all DW images to the corresponding unweighted b0 image.

Input Data: The DW images were organized as 5D arrays representing spatial dimensions, slice number, b-values, and diffusion directions. These were pre-processed and prepared for registration.

Registration Process: For each slice:

- The b0 image was used as the reference (fixed image).
- A body mask was created using Otsu thresholding
- Each of the DW images (from different b-values and directions) was registered to this reference.
- The registration was performed in two steps: a rigid transformation to align overall position and orientation, followed by a B-spline transformation to correct finer distortions.
- The final transformation was applied to the moving image to generate the corrected version.

To evaluate the effect of registration, we measured image similarity using mutual information and structural similarity (SSIM) before and after registration. Registering one slice (including 3 b-values and 3 directions) took around 200 seconds on our workstation.

## B-1: Mask Generation

The input image was first smoothed using Gaussian blurring to reduce noise. We then used Otsu’s method to create an initial binary mask, which was refined by applying morphological opening and closing operations (using a 2-pixel radius) to remove small imperfections and fill any gaps. This final mask was applied to the reference image to ensure consistent and focused segmentation of the relevant region [Figure S2].


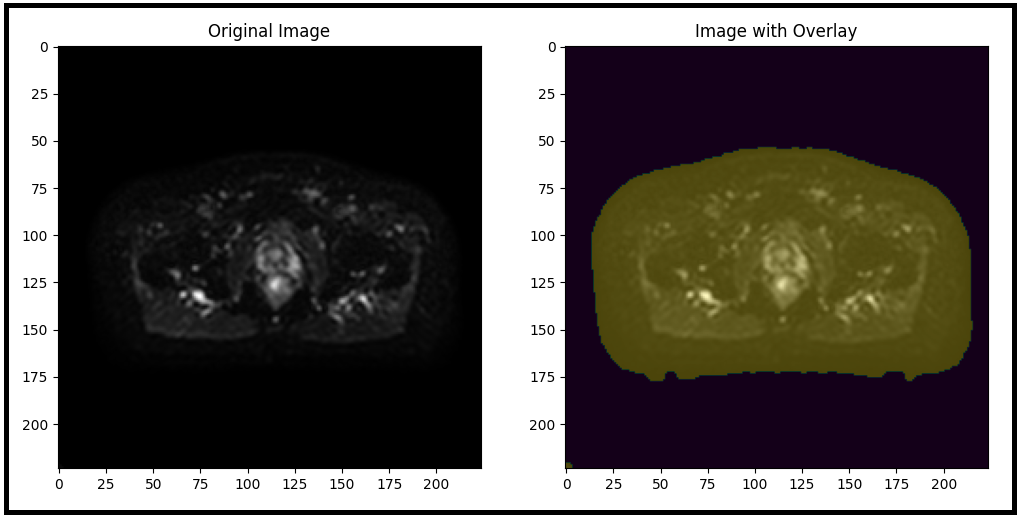


Figure S2: Visualization of the fixed b0 image and its corresponding mask obtained through Otsu thresholding. The original b0 image is displayed in grayscale [left], the generated mask is overlayed on the image [right].

## B-2: B-Spline registration

The B-spline registration was designed for fine alignment and used a multi-resolution approach with three levels of image pyramids. This allowed the registration to progressively refine alignment from coarse to fine, following a pyramid schedule of (4 4 4, 2 2 2, 1 1 1). A B-spline transform was applied to model non-linear deformations, with the control grid spacing set to 10.0 mm. Image similarity was evaluated using Advanced Mattes Mutual Information, along with a rigidity penalty to prevent anatomically unrealistic deformations. Optimization was carried out using Adaptive Stochastic Gradient Descent, running for 200 iterations and sampling 65,536 random points at each step. To preserve image structure, a cubic B-spline interpolator was used to smoothly resample the moving image.

C: Statistical Power Analysis for ADC Bias and Repeatability

## C1: Normality and Effect Size

To evaluate the appropriateness of paired *t*-tests between sequences, we first assessed the normality of the paired differences, using the Shapiro-Wilk test. Differences were computed between EPI Z and EPI, based on the 12 paired ADC measurements presented in Table S4.

Table S4. ADC Values and Paired Differences (EPI Z − EPI)

| Scan | EPI | SPLICE | SPLICE LH | EPI Z | Difference (EPI Z − EPI) |
| --- | --- | --- | --- | --- | --- |
| 1A.1 | 1238.00 | 1304.00 | 1322.00 | 1687.00 | 449.00 |
| 1A.2 | 1317.50 | 1283.50 | 1457.50 | 1875.00 | 557.50 |
| 1B.1 | 1273.50 | 1242.00 | 1303.00 | 1404.50 | 131.00 |
| 1B.2 | 1262.50 | 1319.00 | 1428.50 | 1524.50 | 262.00 |
| 2A.1 | 1257.00 | 1280.75 | 1436.50 | 1970.25 | 713.25 |
| 2A.2 | 1280.00 | 1317.00 | 1310.25 | 1825.00 | 545.00 |
| 2B.1 | 1287.00 | 1315.25 | 1462.75 | 2059.75 | 772.75 |
| 2B.2 | 1179.75 | 1350.25 | 1442.00 | 1993.50 | 813.75 |
| 3A.1 | 1390.80 | 1345.80 | 1399.40 | 1917.40 | 526.60 |
| 3A.2 | 1422.80 | 1470.20 | 1544.20 | 1862.80 | 440.00 |
| 3B.1 | 1423.00 | 1442.50 | 1489.75 | 2020.50 | 597.50 |
| 3B.2 | 1427.00 | 1513.25 | 1525.25 | 1867.50 | 440.50 |

The Shapiro-Wilk test on the paired differences (EPI Z − EPI), performed in MATLAB using the swtest.m function [1], yielded a W-statistic of 0.959 and a p-value of 0.776, indicating no significant deviation from normality. Therefore, the assumptions for applying a paired *t*-test are satisfied.

Similarly the W-statistic and p-values for the other comparisons are given in Table S5.

Table S5: Shapiro-Wilk Normality Test on Paired Differences

| Comparison | W-statistic | p-value | Normality Assumption |
| --- | --- | --- | --- |
| SPLICE vs EPI | 0.919 | 0.236 | Not rejected |
| SPLICE_LH vs EPI | 0.962 | 0.810 | Not rejected |
| EPI Z vs EPI | 0.959 | 0.776 | Not rejected |

Effect sizes were computed using Cohen’s *d*. Post hoc power was calculated in G*Power 3.1.9.7 [Faul, F., Erdfelder, E., Buchner, A., & Lang, A.-G. (2023). G*Power (Version 3.1.9.7) Universität Kiel. <https://www.gpower.hhu.de/>] based on these values.

Table S6. Effect Sizes and Statistical Power (vs. EPI)

| Sequence | Mean | SD | Cohen’s *d* | Statistical Power |
| --- | --- | --- | --- | --- |
| EPI | 1313.24 | 82.91 | – | – |
| SPLICE | 1348.63 | 83.07 | 0.43 | 0.275 |
| SPLICE_LH | 1426.76 | 80.14 | 1.39 | 0.992 |
| EPI Z | 1833.98 | 200.52 | 3.39 | 1.000 |

## C2: Repeatability Power Estimation

To assess the adequacy of six repeated scans for evaluating repeatability, we performed a Monte Carlo simulation-based power analysis using the Repeatability Coefficient (RC) framework:

*RC = 1.96 × √2 × wsd (within-subject standard deviation)*

This coefficient defines the expected range within which 95% of repeated measurements for the same subject are expected to fall.

We performed a Monte Carlo simulation‑based power analysis using the Repeatability Coefficient (RC) framework (RC = 1.96 × √2 × wsd), which defines the range within which 95% of repeated measurements for the same subject are expected to fall. In these comparisons, we specifically ask whether the DW‑EPI sequence demonstrates superior repeatability relative to each of the other protocols serving as references (Tables S7 and S8), and whether registration (under test) improves repeatability vs. regular workflow (reference) for the mentioned contours (Table S9). To do this, we simulated 10,000 repetitions of within‑subject differences based on the estimated true RC and computed the proportion of instances where the observed RC was lower than the reference RC threshold (derived from the comparator method). This approach also allowed us to determine the number of repeated scans required to reliably detect an improvement in repeatability (>80% power) if not already met.

Volunteer Data Results

Table S7. Short-Term Repeatability power statistics (DW-EPI RC = 112 ×10^-6^ mm^2^/s)

| Compared Against | Reference RC (×10⁻⁶ mm²/s) | **Estimated Power at nPair = 6 @ alpha = 0.05** | Minimum repeat pairs needed for ≥ 80% power |
| --- | --- | --- | --- |
| DW-SPLICE | 137 | 0.81 | Sufficiently powered |
| DW-SPLICE_LH | 216 | 0.99 | Sufficiently powered |
| DW-EPI Z | 255 | 0.99 | Sufficiently powered |

Table S8: Long-Term Repeatability power statistics (DW-EPI RC = 97 ×10^-6^ mm^2^/s)

| Compared Against | Reference RC  (×10^-6^ mm^2^/s) | **Estimated Power at nPair = 6 @ alpha = 0.05** | Minimum repeat pairs needed for ≥ 80% power |
| --- | --- | --- | --- |
| DW-SPLICE | 109 | 0.72 | 19 |
| DW-SPLICE_LH | 150 | 0.96 | Sufficiently powered |
| DW-EPI Z | 601 | 0.99 | Sufficiently powered |

Patient Data Results

We extended the repeatability analysis to the patient dataset, comparing registered vs. reference workflows across contours.

Table S9. Patient Repeatability Power statistics

| Contour | Registered RC | Reference RC  (×10^-6^ mm^2^/s) | **Estimated Power at nPair = 7 @ alpha = 0.05** | Minimum repeat pairs needed for ≥ 80% power |
| --- | --- | --- | --- | --- |
| GTV | 438 | 483 | 0.71 | 28 |
| NT-P | 251 | 362 | 0.94 | Sufficiently powered |
| WP | 200 | 234 | 0.77 | 10 |

D: Impact of Registration on sequences for volunteer and phantom experiments


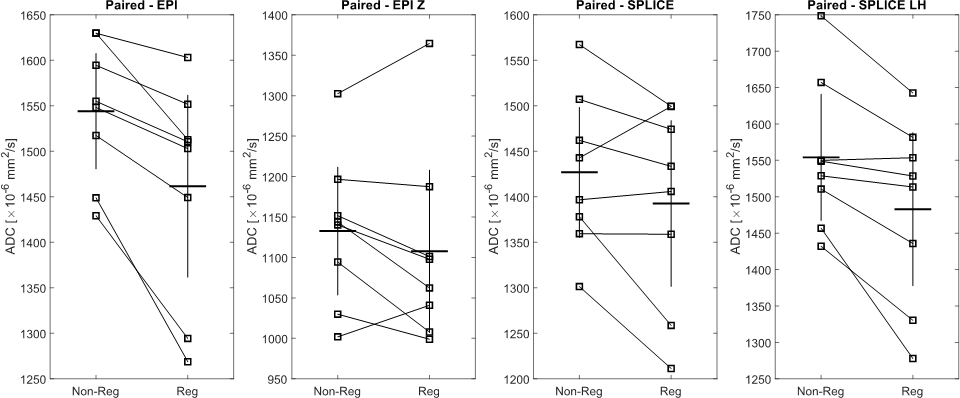


Figure S3: Paired ADC (N = 8, from subjects where raw data was saved) obtained from non-registered (Non-reg) and registered (Reg) workflows. Overall, we found a trend that registration reduced the ADC. Since the vendor’s ADC calculation could not be applied to these data, values differ slightly from Fig. 3.

Table S10: Mean difference between the non-registered and registered ADC pairs for the measured sequences in two volunteer subjects (N = 8 scans each). A p < 0.05 was considered significant. The difference was not significant for SPLICE and EPI-Z sequences.

|  | Mean ADC difference [Non-registered – registered] | P-value |
| --- | --- | --- |
| EPI | 84.42 | 0.003* |
| EPI Z | 25 | 0.22 |
| SPLICE | 34.21 | 0.13 |
| SPLICE LH | 71.11 | 0.01* |


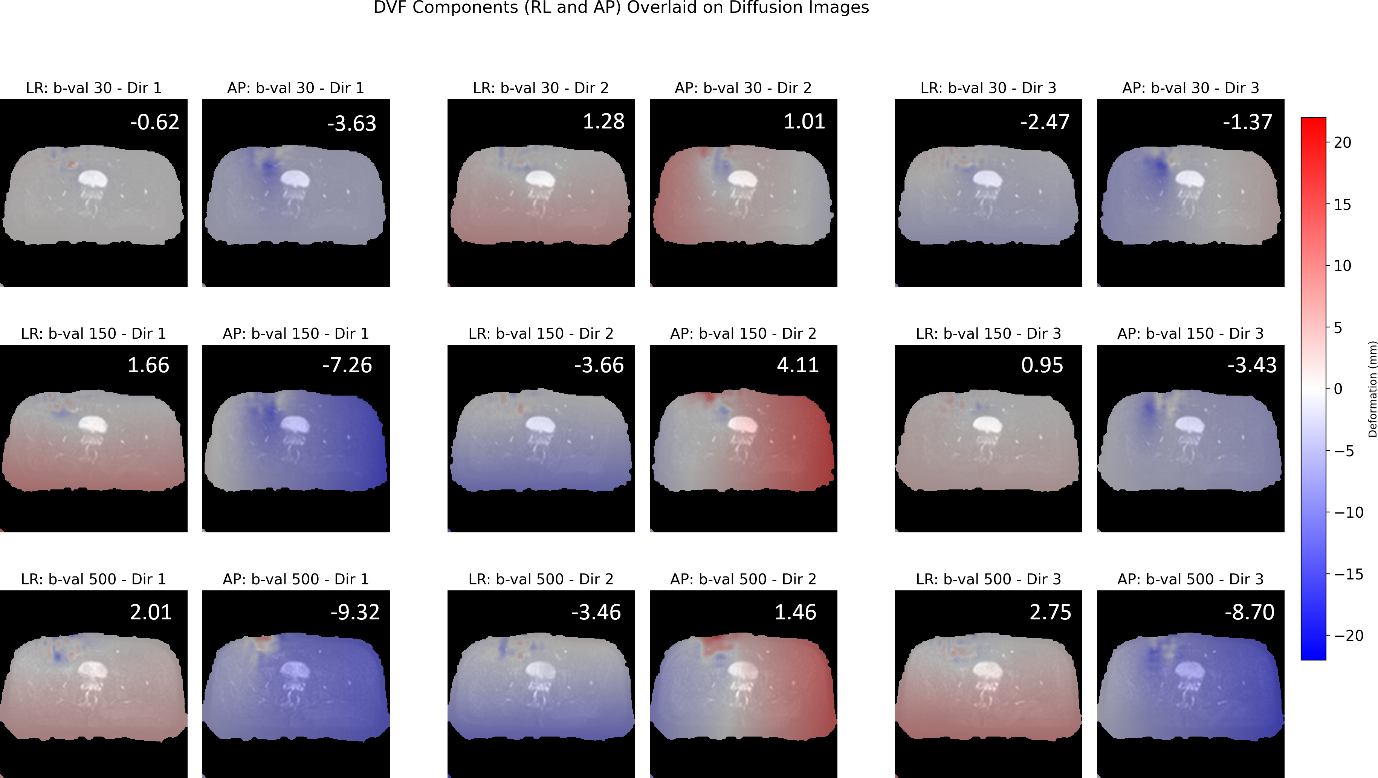


A


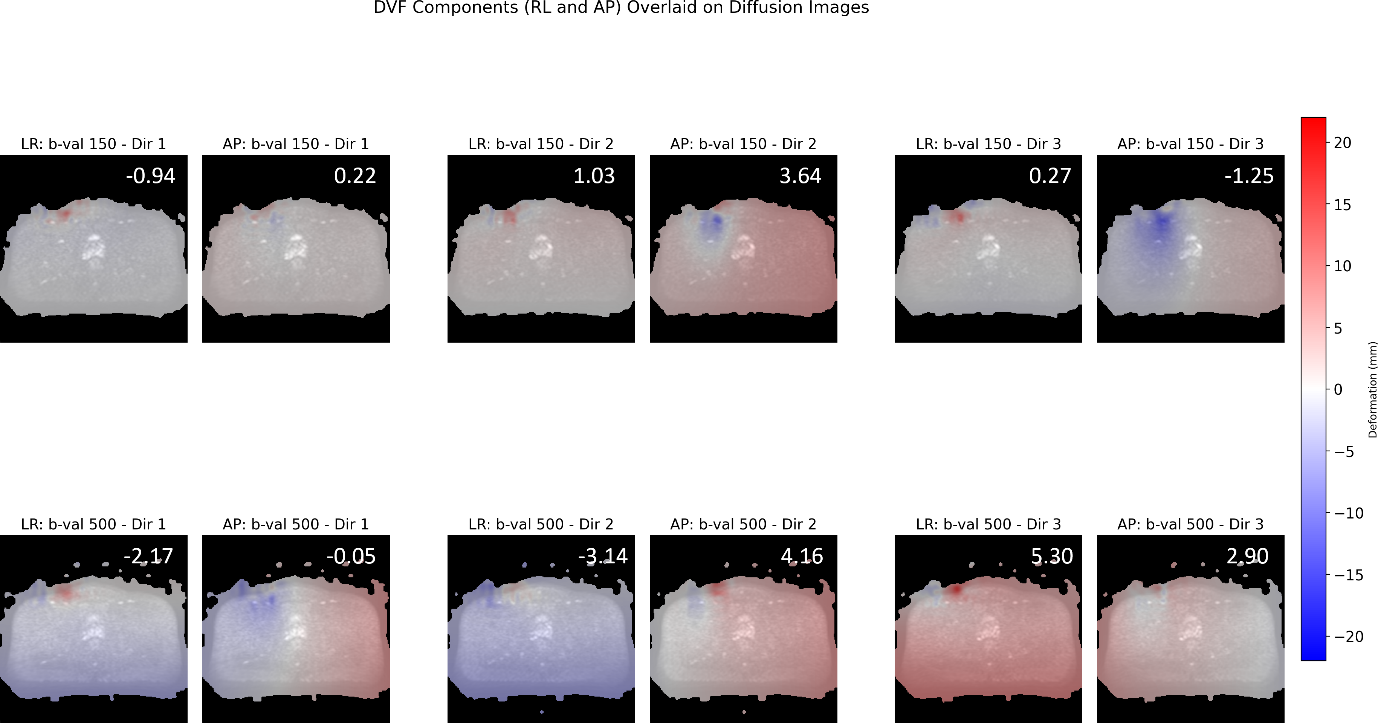


B

Figure S4: Registration results for a slice containing the prostate ROI. Deformation fields in the left–right (LR) and anterior–posterior (AP) directions for each b-value and diffusion encoding direction (against undeformed b0) are overlaid onto the corresponding DW image. Numbers indicate the average deformation (mm) within the prostate. For each DW direction, the scanner’s three physical axes (X, Y, Z) contribute: Dir 1 = [-0.67, 0.33, -0.67], Dir 2 = [0.33, -0.67, -0.67] and Dir 3 = [-0.67, -0.67, 0.33]. In our example, deformation is generally higher for the DW-EPI sequence (A) than for the DW-SPLICE sequence (B).

Table S11: SNR (b0) and RC at the central vial of the QIBA phantom for the different sequences. There is no change in SNR and we observed only small changes in RC.

| Sequence | Reg/NR | SNR | RC (10^-6^ mm^2^/s) |
| --- | --- | --- | --- |
| EPI | NR | 65 | 11.12 |
|  | Reg | 65 | 11.08 |
| EPI-AP | NR | 95 | 4.54 |
|  | Reg | 95 | 5.46 |
| EPI-Z | NR | 69 | 17.93 |
|  | Reg | 69 | 18.69 |
| SPLICE | NR | 43 | 52.15 |
|  | Reg | 43 | 51.73 |
| SPLICE-LH | NR | 37 | 68.91 |
|  | Reg | 37 | 64.94 |

Across all 13 vials, the mean ADC (paired NR vs Reg) showed no significant difference for any sequence (all *p* > 0.05). Overall, we found no significant impact of registration for the QIBA phantom data analysis.

E: Sample Phantom and volunteer images for SNR comparison


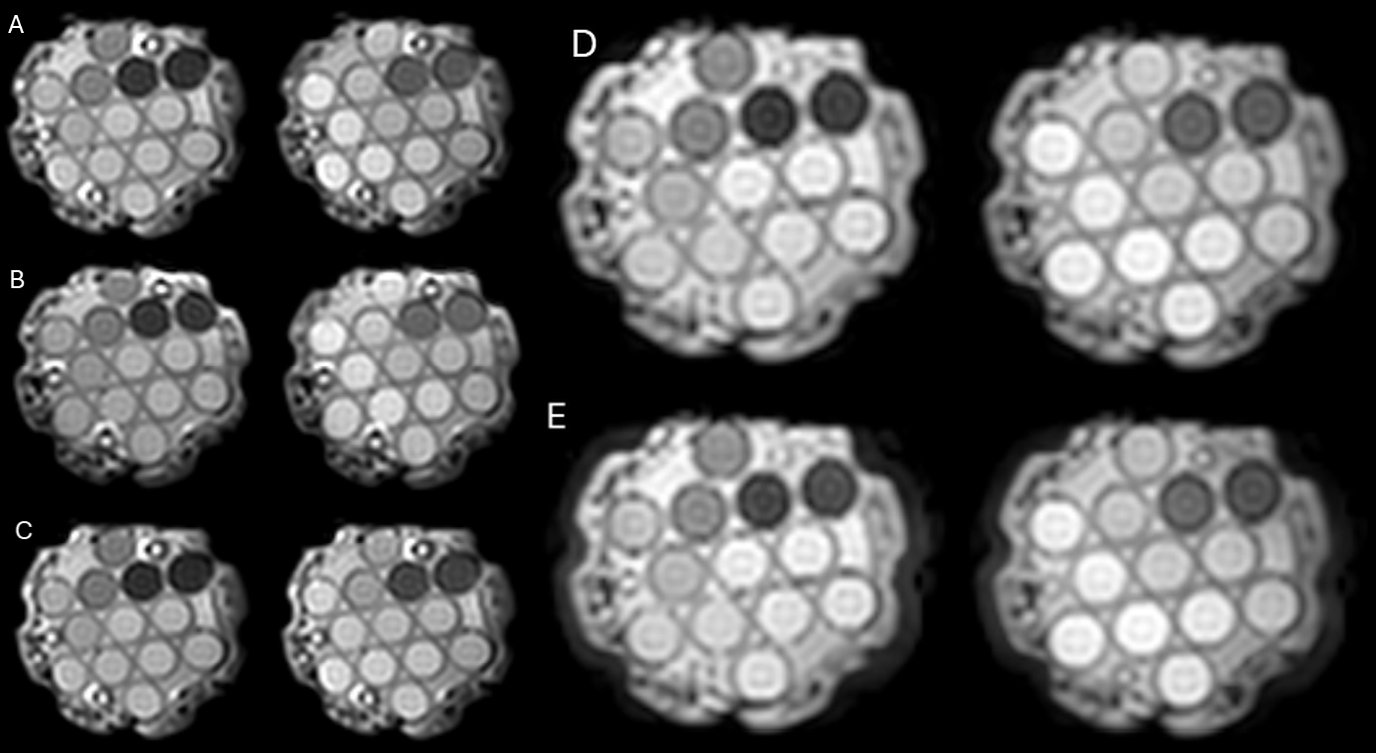


Figure S5: Diffusion-weighted images at b0 (left) and b500 (right). (A) DW-EPI, (B) DW-EPI AP, (C) DWI-Z, (D) DW-SPLICE, and (E) SPLICE LH.


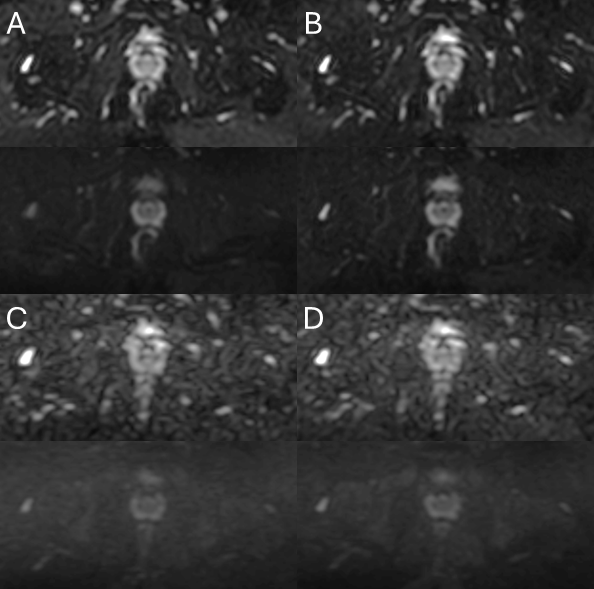


Figure S6: Representative volunteer scan showing diffusion-weighted (trace) images at b0 (top row) and b500 (bottom row). (A) DW-EPI, (B) DW-EPI Z, (C) DW-SPLICE, and (D) SPLICE LH.

References:

1. Ahmed BenSaïda (2025). Shapiro-Wilk and Shapiro-Francia normality tests. (https://www.mathworks.com/matlabcentral/fileexchange/13964-shapiro-wilk-and-shapiro-francia-normality-tests), MATLAB Central File Exchange. Retrieved May 16, 2025.
